# Identification of IgE-binding peptide and critical amino acids of *Jatropha curcas* allergen involved in allergenic response

**DOI:** 10.1186/s40064-016-2036-5

**Published:** 2016-04-14

**Authors:** Livia Maia Crespo, Natalia Deus de Oliveira, Renato Augusto Damatta, Viviane Veiga do Nascimento, Thais Pacheco Soares, Olga Lima Tavares Machado

**Affiliations:** Laboratório de Química e Função de Proteínas e Peptídeos, Centro de Biociências e Biotecnologia, Universidade Estadual do Norte Fluminense Darcy Ribeiro, Av. Alberto Lamego, 2000, Parque Califórnia, Rio de Janeiro, CEP 28013-602 Brazil; Laboratório de Biologia Celular e Tecidual, Centro de Biociências e Biotecnologia, Universidade Estadual do Norte Fluminense Darcy Ribeiro, Rio de Janeiro, CEP 28013-602 Brazil

**Keywords:** *Jatropha curcas*, Allergen, Biofuel, 2S albumin, IgE-binding peptide

## Abstract

Increasing energy demand has spurred interest in the use of biofuels. *Jatropha curcas* (physic nut), an inedible oilseed, is a potential source of bioenergy. The seeds, however, contain allergens such as Jat c 1, a 2S albumin that can induce hypersensitivity reactions in humans and result in allergic diseases. Recent advances in identifying and characterizing plant allergens and, in particular, their immunoglobulin E (IgE)-binding epitopes have produced a wealth of information. We identified IgE-binding regions and the critical amino acids involved in the degranulation of mast cells and the release of histamine, preliminary steps for the prevention and treatment of this allergy. Four IgE-binding regions were identified in the sequence of Jat c 1. We identified and demonstrated the fundamental role of two glutamic acid residues in IgE binding. The sequence LEKQLEEGEVGS produces a random loop on the most exposed part of Jat c 1. This region is important to the stimulation of the allergic response. The possibility of using this information to produce vaccines and other pharmacological agents for allergy treatment is discussed.

**Electronic supplementary material:**

The online version of this article (doi:10.1186/s40064-016-2036-5) contains supplementary material, which is available to authorized users.

## Background

The increasing demand for food and fuel as lifestyle expectations rise in a population expected to reach nine billion by 2050 will soon become global challenges (Cohen et al. 2005; Lutz and Samir 2010). Food and fuel security are inextricably linked. Indeed, the competition between the use of oilseeds as biodiesel and food is increasing. Inedible oilseeds, such as those that contain toxins and allergens, may thus represent an ideal choice of feedstock for the biofuel industry.

*Jatropha curcas* is an oleaginous plant able to grow under various agroclimatic conditions and on land with thin soil cover (Devappa et al. 2010, 2011). It is widely grown in Mexico, Nicaragua, northeastern Thailand and in parts of India and is being promoted in southern Africa, Brazil, Mali and Nepal. Several governments, international organizations and national bodies are promoting the planting and use of *J. curcas* and other oil-bearing plants as biofuels (Openshaw 2000; Makkar et al. 2009). Studies are being developed to maximizing the production of biofuel with the direct use of the *J. curcas* oil (Go et al. 2016). *J. curcas* is superficially a promising oilseed because of its high oil content and its inedibility, due to its high toxicity (Makkar et al. 2009). The toxic genotype is prevalent throughout the world and the non-toxic genotypes exist only to the Mexico that is genetically differentiated (Massimo et al. 2015). This varieties genetically improved are being investigated by the technology of DNA-based molecular markers (Chavan and Gaur 2015). These toxic and allergenic factors (Maciel et al. 2009), however, have also limited its use in biofuel production, because the toxins restrict the use of the cake, and the allergens compromise the safe handling of the seeds.

The elucidation of the primary and three-dimensional structures of allergens, including the identification of regions involved in allergic reactions, such as IgE-binding, B cell and T-cell epitopes, is critical to the understanding of the allergic mechanisms elicited by these proteins and the possible cross-reactions between different allergens. Such identification allows the development of a panel of allergenic epitopes, identifying the common aspects among these epitopes, and can direct the development of specific immunotherapies that are effective against a group of cross-allergens. Vaccines based on epitopes may thus avoid some of the problems with the vaccines developed from plant extracts or from whole proteins. Jat c 1, which cross-reacts with the *Ricinus communis* allergen, is the only allergenic protein yet isolated from *J. curcas* seeds (Maciel et al. 2009). Maciel et al. (2009), however, only described the N-terminus of Jat c 1, which prevented the elucidation of its allergenic epitopes.

We have thus purified and fully characterized Jat c 1, identified regions involved in allergenic response and searched for homologous IgE-binding epitopes in allergenic proteins from other plants. The results presented herein increase the information available for this *J. curcas* allergen and may contribute to future efforts at developing immunotherapeutic and allergen-inactivation strategies to ensure that its oil extraction is safe for biofuel production.

## Methods

### Investigation of sequencial IgE-binding epitopes: denaturation, reduction and alkylation

*Jatropha curcas* seeds were obtained from EMBRAPA (Empresa Brasileira de Pesquisa Agropecuária), Brazil, and Jat c 1 was isolated and identified by SDS-PAGE and immunoblotting as described by Maciel et al. (2009). The molecular weight of the isolated protein was determined by mass spectrometry using a Synapt G2SI Waters spectrometer. Jat c 1 was denatured with 6 M guanidinium chloride, reduced with 2 mM dithiothreitol and alkylated with 4-vinylpyridine (560 μmol), as described by Felix et al. (2008), for investigating the presence of continuous epitopes. The reaction mixture was submitted to C18 reverse-phase HPLC for *S*- and *L*-chain separation. The chromatograph had a flow rate of 0.7 mL min^−1^ using 0.1 % TFA as solvent A and 80 % acetonitrile containing 0.1 % TFA as solvent B. The gradient was 0–80 % of solvent B for 55 min. Proteins were detected by monitoring the absorbance at 220 nm. The N-terminal amino acid sequences of both the large and small isolated chains were obtained on a Shimadzu PPSQ-33 automated protein sequencer. PTH amino acids were detected after separation on a reverse-phase C18 column under isocratic conditions, following the manufacturer’s instructions. The polypeptide sequences obtained were aligned automatically with a BLAST search. The allergenic properties of each isolated chain were investigated by a mast-cell degranulation assay as described below.

### Animals and antiserum

#### Animals and production of polyclonal antibodies

Isogenic female R/A Tor rats 70 days old weighing 150 g, considered to be good producers of IgE, were obtained from the animal house of the Universidade Federal Fluminense, Niteroi, RJ, and all experimental procedures were approved by the animal research ethics committee (Protocol 112).

Jat c 1 (10 mg) in 500 µL of sterile 0.01 M phosphate-buffered saline (PBS) was emulsified with an equal volume of an adjuvant containing 5 mg of Al_2_OH_3_ and 5 mg of nigrosin. This mixture was injected into the four foot pads of the animals (250 µL/foot pad). Five rats were anesthetized after 15 days and bled by cardiac puncture, and the titer was determined by indirect competitive ELISA. The sera were pooled, lyophilized and stored at −20 °C until used in the mast-cell degranulation assay.

#### ELISA: monitoring antibody production

Microtiter-plate wells were each coated with 100 µL of 1 mg/mL Jat c 1 in 0.2 M sodium carbonate buffer, pH 9.6, and incubated for 18 h at 37 °C. The subsequent steps were performed at 37 °C. A microtiter plate was washed twice with PBS containing 0.05 % TWEEN 20 (300 µL/well for 1 h), 300 µL of blocking buffer (1 % gelatin in PBS containing TWEEN 20) were added to each well and the plate was incubated for 1 h. The plate was again washed with PBS containing 0.05 % TWEEN (300 µL/well for 1 h). After coating with Jat c c1, each well received 50 µL of antiserum diluted 1:500 in PBS containing 0.05 % TWEEN 20. IgE was detected by a 1:2000 dilution of goat anti-rat immunoglobulin (anti IgE) conjugated to peroxidase (Sigma) developed in 50 µL of a solution containing 10 mg OPD, 10 µL of 30 % H_2_O_2_, 6.5 ml of 0.1 M citric acid, 7.0 ml of 0.2 M sodium phosphate and 9.0 mL of distilled H_2_O. The reaction was stopped by adding 50 µL of H_2_SO_4_ per well. The plate was read at 492 nm.

Two blanks were included in the experiment. Blank 1 contained protein and the development solution to detect any intrinsic peroxidase activity in the samples, and Blank 2 contained carbonate/bicarbonate buffer, antibody and the development solution.

### Mast-cell degranulation assay

#### Rat peritoneal mast cells

Wistar rats were bred at the animal house of the Universidade Estadual do Norte Fluminense. All experimental procedures were approved by the university’s animal research ethics board (Protocol 112).

For obtaining peritoneal mast cells, three rats (250 ± 20 g) were individually euthanized with CO_2_. The abdomens were gently massaged for approximately 90 s, and then an incision approximately 5 cm in length was cut in the peritoneal cavities, which were carefully opened. The cavities were washed prior to the injection of 20 mL of DMEM (Gibco) containing 12 U/mL of heparin, as described by Deus-de-Oliveira et al. (2011). The fluid containing peritoneal cells was aspirated with a Pasteur pipette. The fluid from the first rat was used for washing the second, and so on, to enrich the content of the wash with mast cells. The final content of the collected peritoneal fluid, approximately 15 mL, was transferred to Petri plates and incubated for 30 min at 37 °C to separate the mast cells from the macrophages. Two-thirds of the upper layer were then aspirated and discarded. The supernatant (approximately 4–5 mL) enriched with mast cells (~1.8 × 10^5^ mast cells/mL) was separated into aliquots of 100 µL in Eppendorf tubes.

#### Mast-cell activation

The mast cells (100 µL) were incubated with 1 μL of R/A Tor rat pre-immune serum as a control or were initially sensitized for 1 h at 37 °C using 1 μL of anti-Jat c 1 polyclonal antibodies. After sensitization, the mast cells were washed twice with Dulbecco’s Modified Eagle Medium (DMEM) (Sigma) and were then incubated with 100 ng of synthetic peptides or 100 ng of native Jat c 1 or its isolated chains (100 ng) for 1 h at 37 °C. To evaluate the mast-cell degranulation, we mixed and incubated an aliquot of 10 µL of the cell suspension, after the various incubations, for 15 min with 10 µL of an aqueous solution containing 0.1 % toluidine blue, 10 % formaldehyde and 1 % acetic acid, pH 2.8. The intact and degranulated mast cells were counted in a Neubauer chamber. The four quadrants were microscopically viewed using Nomarski differential interference contrast with a Zeiss Axioplan optical microscope. Untreated mast cells were also incubated as a negative control with the dye under the above conditions and were observed with the optical microscope. The counting of intact and degranulated cells allowed an assessment of the acquisition procedure. Each experiment was performed in triplicate using mast cells from the same animals. Graphs of the results of the degranulation assays were constructed using GraphPad Prism.

The data obtained in the degranulation assays were analyzed by one-way ANOVAs and multiple comparisons using Tukey’s tests (*P* < 0.001). This analysis was performed by GraphPad Prism.

### Synthesis of peptides for identifying IgE-binding regions

Twelve peptides, 9–24 AA residues in length and based on the sequence of Jat c 1, were synthesized for mapping and characterizing IgE-binding regions using the multiple method of the 9-fluorenylmethoxycarbonyl (Fmoc) (Sigma) strategy (Ruiter et al. 2006). The amino acid sequences of the synthetic peptides are presented in Additional file 1: Figure S1).

Each peptide was probed individually with mast cells sensitized with rat IgE serum (immunized with Jat c 1), and mast-cell degranulation was evaluated as described below. A peptide was considered to be an IgE-binding region at levels of mast-cell degranulation >40 % (Felix et al. 2008).

### Identification of amino acids involved in IgE binding

We used two strategies to identify the amino acids that bind to IgE pre-fixed in mast-cells: chemical modification of native synthetic peptides with Woodward’s Reagent K (WRK), and substitution of strategic amino acid residues in the characterized IgE-binding-regions.

#### Modification with Woodward’s Reagent K

WRK (*N*-ethyl-5-phenylisoxazolium-3′-sulfonate) (Sigma) is a specific reagent for the modification of dicarboxylic amino acids. The methodology proposed by Dunn et al. (1974) was used with modifications described by Deus-de-Oliveira et al. (2011). Synthetic peptides were treated with 250 mM WRK and incubated at 35 °C for 3 h. The chemical modification was evaluated by reverse-phase HPLC and mass spectrometry. The allergenicity of the treated peptides was analyzed by mast-cell degranulation assays as previously described.

#### Substitution of strategic amino acid residues

The AA sequences of the IgE-binding peptides of Jat c 1 contained two or more dicarboxylic residues (glutamic acid or aspartic acid). We determined if these residues were critical in these epitopes by constructing a library of peptides substituting glutamic acid by leucine using the Fmoc strategy described above. All peptides were purified by C18 reverse-phase HPLC. The IgE binding of mutated peptides was analyzed by a mast-cell degranulation assay as previously described.

### Structural studies

IgE-epitope candidates were localized using JHg, modeled by Nair et al. (2012). The model of the processed Jat c 1 was constructed with Swiss-PDB Viewer. The structure of Jat c 1 was also modeled after substitution of some residues of aspartic acid and glutamic acid by leucine using the same program, and this structure was evaluated by PROCHECK.

## Results

We purified and fully characterized Jat c 1, a known allergenic protein from *J. curcas* seeds. We also identified IgE binding-regions of Jat c 1 and searched for homologous sequences in allergenic proteins from other plants that trigger allergenic cross-reactions.

### Isolation and characterization of Jat c 1

**Fig. 1:**
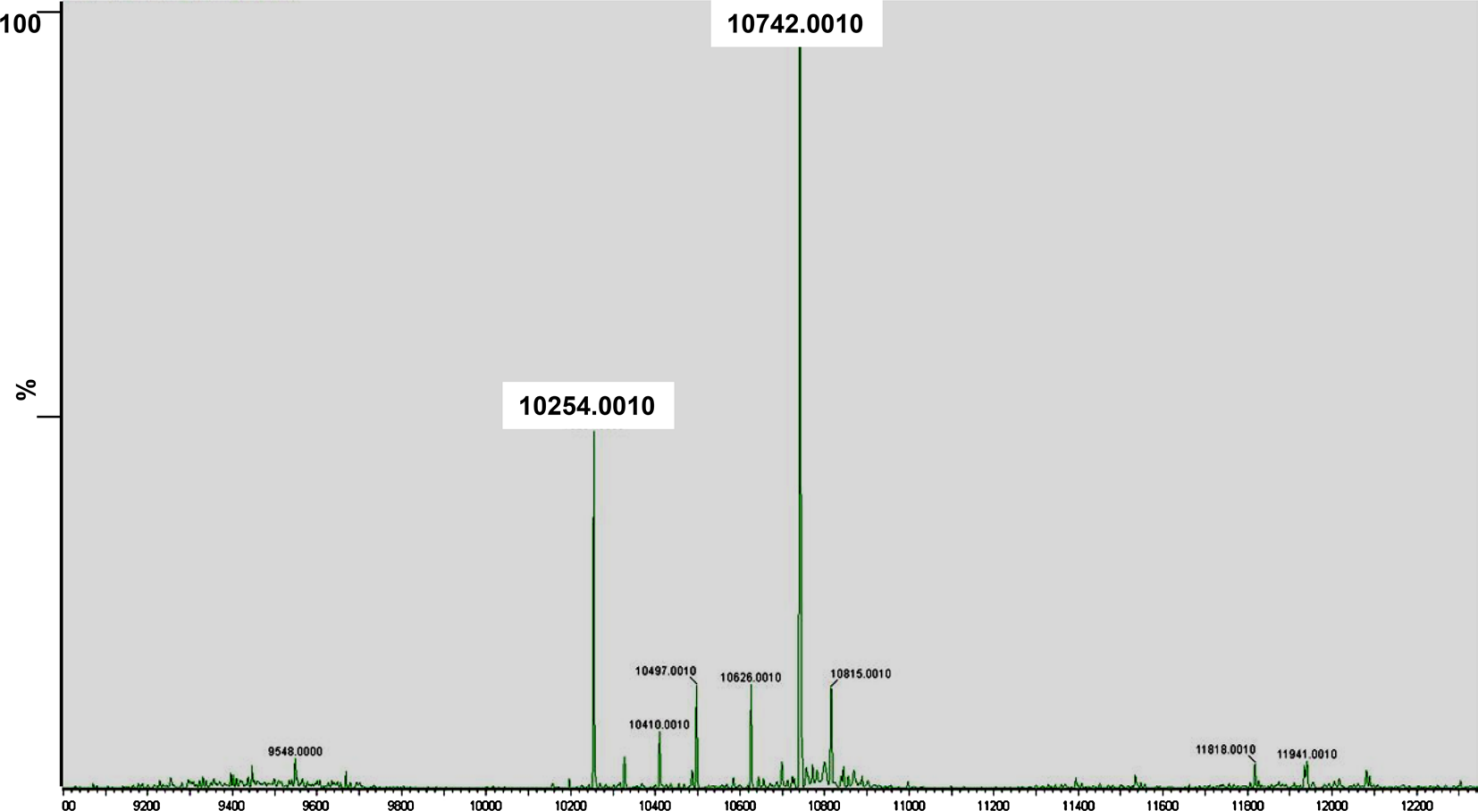
Mass spectrum of Jat c 1, an allergenic protein from *Jatropha curcas*. Molecular weight of native Jat c 1 measured on Synapt G2 SI

The 2S albumin fraction from *J. curcas* seeds was obtained by saline extraction and chromatography on Sephadex G-50. Jat c 1 was then isolated by reverse-phase chromatography, as previously reported (Maciel et al. 2009). Mass spectrometry identified two proteins of 10.254 and 10.742 kDa (Fig. 1).

**Fig. 2:**
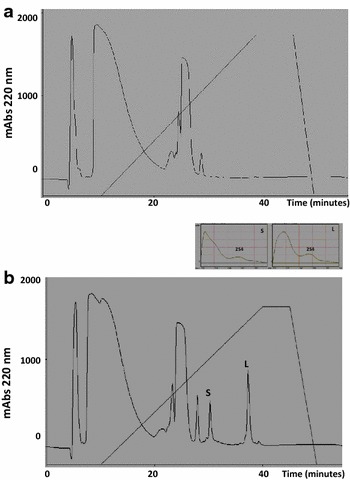
Reversed-phase chromatographic isolation of polypeptide chains from Jat c 1. Jat c 1 was treated with guanidine chloride, DTT and vinyl pyridine and was fractionated on a C18 column. **a** Blank reaction. **b** Elution profile of the Jat c 1 polypeptide chains after denaturation. *Peak S* (small chain) and *Peak L* (large chain). Elution conditions: solvent A, 0.1 % TFA; solvent B, 80 % acetonitrile/0.1 % TFA. The elution profile was monitored at 220 nm, and the *oblique line* represents the acetonitrile gradient. *Insets* in **b**. Spectra of Jat c 1 small (*S*) and large (*L*) chains

After the reduction and pyridylethylation of Jat c 1 (10.742 kDa), two polypeptide chains were isolated by reverse-phase chromatography on a C18 column (Fig. 2b). The UV spectra of both chains peaked at 254 nm, indicating the production of a pyridylethyl cysteine after the reduction and alkylation (Fig. 2b insets S and L).

**Fig. 3:**
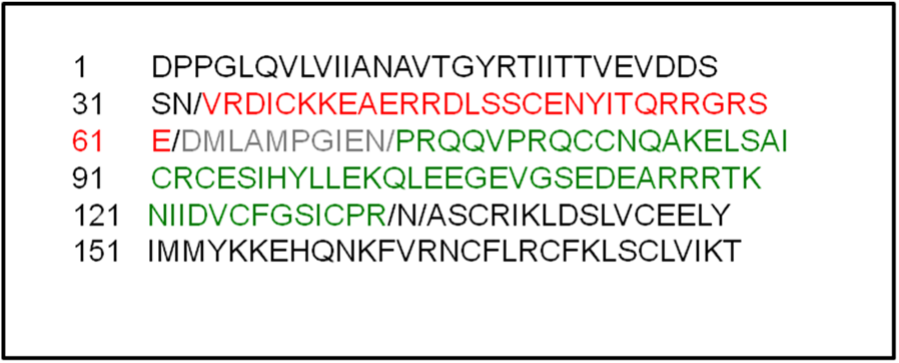
BLAST alignment of the N-terminal sequence of Jat c 1 with the NCBI GenBank database. Both chains aligned with hypothetical protein JCGZ_00819 from *Jatropha curcas* at positions 33–61 for the small chain (*red*) and 72–103 for the N-terminal partial sequence of the large chain (*green*). The linked peptide (62–71) is in *gray*. Based on the molecular weight determined by mass spectrometry, the large chain continued to position 133. The symbol “*slash*” indicates possible regions of precursor processing

The N-terminal sequences of the first (small chain) and second (large chain) Jac c 1 peaks were VRDICKKEAERRVLSSCENTITQRRGRSE and PRQQVPRQCCNQAKELSAICRCESIHYLLEKQ, respectively. Alignment and analysis of these sequences were obtained from the Jatropha Genome Database (http://www.kazusa.or.jp/jatropha/). The small chain aligned at position 33–61, and the large chain (N-terminal partial sequence) aligned at position 72–103. The molecular weight determined by mass spectrometry suggested that the large chain continued to position 133 (Fig. 3).

### Investigation of continuous epitopes

**Fig. 4:**
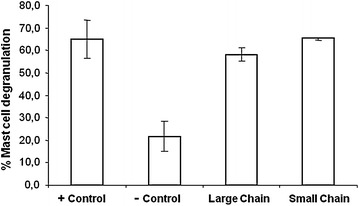
Mast-cell degranulation assay. +Control: mast cells sensitized by Jat c 1 antiserum and incubated with Jat c 1; −Control: mast cells incubated with DMEM; large chain, small chain: mast cells sensitized by Jat c 1 antiserum and incubated with the large or small chain, respectively, isolated from Jat c 1 under denaturation, reduction and alkylation conditions. Mean values from 5 experiments ± SD are presented. Statistical analysis was performed using one-way ANOVA followed by Turkey’s test. *p < 0.001

Mast cells sensitized with rat-serum IgE and subsequently challenged with native Jat c 1 (+control) showed approximately 60 % degranulation. Mast cells challenged with isolated Jat c 1 polypeptide chains produced similar results. The small and large chains produced 58 and 64 % degranulation, respectively, indicating the presence of continuous IgE epitopes in both chains (Fig. 4).

**Fig. 5:**
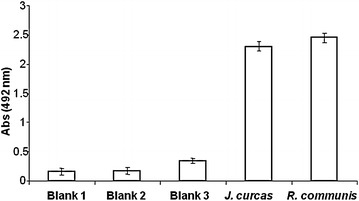
ELISA for IgE anti-Jat c 1 antibodies. Sera from rats immunized with Jat c 1 were assayed for Jat c 1 (control) and allergens from *R. communis*. Blanks 1, 2 and 3 contained water, PBS and pre-immune serum, respectively. IgE anti-Jat c 1 recognized a mixture of Ric c 1 and Ric c 3. Mean values from 5 experiments ± SD are presented. Statistical analysis was performed using one-way ANOVA followed by Turkey’s test. *p < 0.001

**Fig. 6:**
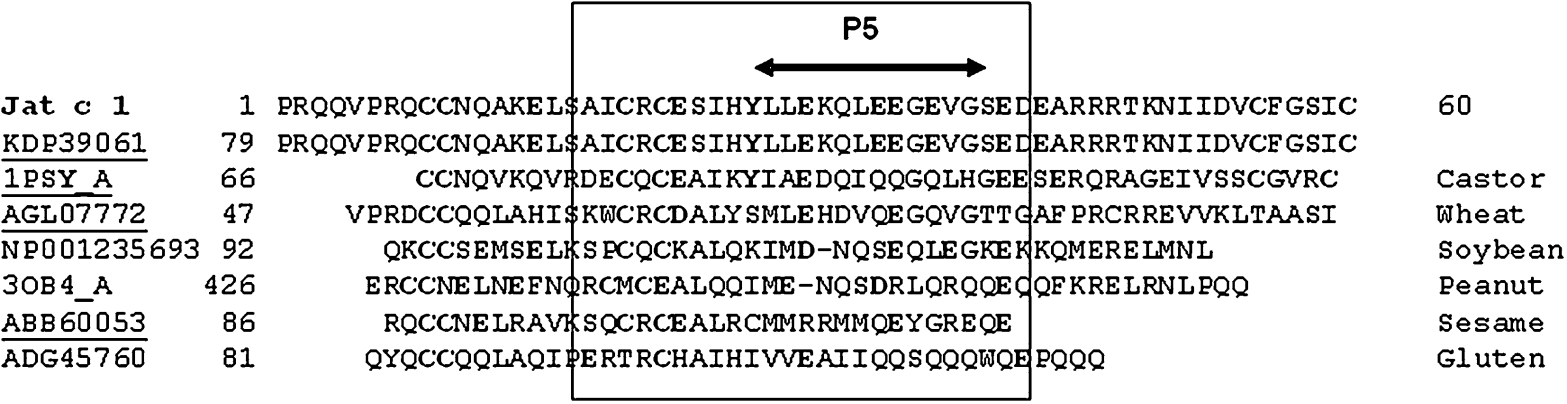
Multiple protein alignment. Query: Jat c 1 large chain; AGL07772—Chain A, 0.19 alpha-amylase inhibitor from wheat [*Triticum aestivum*]; NP001235693—napin-type 2S albumin 1 precursor from soybean [*Glycine max*] 3OB4_A Chain A, Ara H 2 major allergen from peanut [*Arachis hypogaea*]; ABB60053—2S albumin precursor isoform 3 from sesame [*Sesamum indicum*]; ADG45760 avenin-like b from gluten [*Triticum aestivum*]. Some determinant cysteine and glutamic and aspartic acid residues are *highlighted in bold*

We previously reported cross-reactivities of Jat c 1 with allergens from *R. communis* using a passive cutaneous anaphylaxis assay (Maciel et al. 2009). We corroborated this finding using ELISA assays with the native Jat c 1 (Fig. 5). Because the full sequence of Jat c 1 is now known, we performed a BLAST search to find the degree of homology of the large and small chains of Jat c 1 to *R. communis* allergens (Fig. 6). All cysteines and nearly all glutamic acid residues were conserved between Jat c 1 and the *R. communis* allergens.

### Identification of continuous IgE-binding regions

Twelve peptides spanning the entire Jat c 1 sequence were synthesized for investigating the regions corresponding to continuous epitopes (Additional file 1: Figure S1). All synthesized peptides were purified by reverse-phase HPLC (data not shown). Mast cells incubated with pre-immune rat serum and synthetic peptides did not degranulate, indicating that the serum did not recognize any of the synthesized peptides. The peptides P1, P4, P5, P6 and P8 spanning the length of the small and large chains of Jat c 1 were recognized by mast cells sensitized with rat-serum IgE. Mast-cell degranulation was well above 40 % (Table 1). In contrast, mast-cell degranulation was not induced by P2, P3, P7 and peptides P9 to P13, under the same conditions (mast-cell degranulation <40 %).

**Table 1:** Mast cell degranulation induced by synthetic peptide

Synthetic peptide number/amino acid sequences	Mast cell degranulation	Mast cell degranulation by WRK-treated peptide	Amino acid sequences of the peptide were replaced acids glutamic by leucine	Mast cell degranulation by Glu → Leu peptide
Mast cell isolation (negative control)	21.60 ± 6.74	21.60 ± 6.74	–	21.60 ± 6.74
2S albumin (positive control)	65.13 ± 8.5	65.13 ± 8.5	–	65.13 ± 8.5
P1/VR**D**ICKK**E**A**E**RR**D**LS	59.21 ± 6.51	29.76 ± 3.66	VR**L**ICKK**L**A**L**RRD**L**S	43.95 ± 1.48
P4/QAK**E**LSAICRC**E**SIHYL	69.08 ± 3.63	42.35 ± 0.49	QAK**L**LSAICRC**E**SIHYL	38.96 ± 1.86
P5/L**E**KQL**EE**G**E**VGS	71.46 ± 1.41	19.72 ± 1.23	L**L**KQL**EE**G**L**VGS	25.65 ± 5.44
P6/VGS**EDE**ARRRTKNII	66.84 ± 9.76	22.05 ± 1.48	VGS**LDL**ARRRTKNII	32.36 ± 0.50
P8/S**ED**MLAMPGI**E**N	60.95 ± 9.50	30.79 ± 1.11	S**LL**MLAMPGI**E**N	33.17 ± 0.23
P3/PRQQVPRQCCNQAK**E**L	35.08 ± 4.34	24.97 ± 5.17	PRQQVPRQCCN	
P2/SC**E**NYITQRRGR	24.20 ± 2.30	–		
P7/**D**VCFGSICPR	32.06 ± 3.30	–		

Negative control: Mean values from 5 experiments ± SD are presented. Statistical analysis was performed using one**-**way ANOVA followed by Turkey’s test

* p < 0.001

### Characterization of the critical amino acids in the continuous IgE-binding regions

All of the identified continuous Jat c 1 IgE-binding peptides(P1, P4, P5, P6 and P8) contained at least two glutamic acid residues. To verify the possible involvement of these residues in IgE binding, we treated P3 and the synthetic peptides that degranulated mast cells in the previous experiment (P1, P4, P5, P6 and P8) with WRK, a reagent known to modify glutamic acid. After WRK treatment, all peptides except P4 exhibited reduced mast-cell degranulation, with levels similar to those of the negative controls (~30 %). Mast-cell degranulation induced by P4 was 42 %, a value near a negative mast-cell degranulation reported by Deus-de-Oliveira et al. (2011).

To confirm the critical role of glutamic acid in IgE binding, we synthesized a series of related peptides substituting the strategic glutamic acid residues by leucine. The IgE-binding abilities of these modified peptides were evaluated by mast-cell degranulation assays. The substitution of glutamic acid residues by leucine in P5, P6 and P8 significantly reduced mast-cell degranulation to approximately 30 %. Substitution of glutamic acid residues in P1 and P4 decreased mast-cell degranulation to 40 %, similar to that of the negative control (Table 1).

### Structural localization of continuous IgE-binding epitopes

**Fig. 7:**
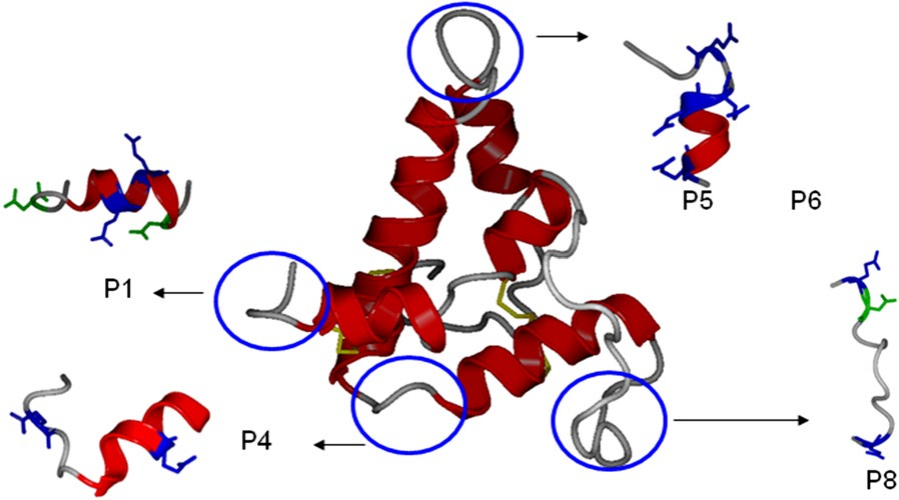
Mapping of the peptides onto a model of the three-dimensional structure of Jat c 1. The structures of IgE-binding peptides are highlighted and located in the Jat c 1 structure

We then localized the identified continuous IgE epitopes (P1, P4, P5, P6 and P8) within the three-dimensional structure of *J. curcas* hemagglutinin (JcCA0234191), a protein homologous to Jat c 1, that was modeled by Nair et al. (2012). P5 and P8 were the most exposed (Fig. 7).

### Sequence alignment and investigation of cross-reactivity between allergens from *R. communis* and *J. curcas*

Because Jat c 1 cross-reacted with allergens from *R. communis* (Maciel et al. 2009), and because *R. communis* allergens can cross-react with allergens from wheat, soybean, peanuts, sesame, gluten and shrimp (Deus-de-Oliveira et al. 2011), we performed a BLAST search of the Jat c 1 large chain with allergen proteins from these food sources. The distribution of cysteine and dicarboxylic residues (glutamic or aspartic acids) was highly conserved in these allergens, particularly the section corresponding to P5 (Fig. 6).

## Discussion

 We previously reported the purification of an allergenic protein from *J. curcas*, Jat c 1, belonging to the 2S albumin family and the prolamin superfamily (Maciel et al. 2009). The cupin and prolamin superfamilies are the most widespread groups of plant proteins that contain allergens (Breiteneder and Radauer 2004). We purified and fully characterized this allergen to better understand the allergenic properties of Jat c 1 and the cross-reactivity of this protein with other allergens. We also identified IgE biding-regions and searched for homologous IgE-binding peptides in allergenic proteins from other foods allergens.

We previously determined the apparent molecular weight of Jat c 1 by SDS-PAGE and described the partial sequence of the N-terminus of this protein (Maciel et al. 2009). The full sequence of Jat c 1 presented in the present study contained slight differences at the initial N-terminal region from the originally isolated allergen, probably because the seeds in the two studies were from different varieties of *J. curcas*. The isolated fraction was subjected to mass spectrometry, which indicated the presence of two proteins of 10.254 and 10.742 kDa.

A hemagglutinin isolated from *J. curcas*, JHg (Nair et al. 2012), is homologous to Jat c 1. These authors speculated that JHg would thus probably have allergenic properties. They proposed a role for the carbohydrate moiety in this allergenic activity. Another homologous protein, JcT-I, MW 10.252 kDa isolated by Costa et al. (2014), is a glycoprotein with an antibacterial effect due to its trypsin inhibitory activity. Alignment of Jat c 1 with JHg and JcT-I indicated that they had the same canonical sequence, so they could be considered “proteoforms”, a term suggested by Smith and Kelleher (2013) to designate all molecular forms of protein products of a single gene, including changes due to genetic variations, alternatively spliced RNA transcripts and post-translational modifications.

The allergic reaction mediated by Jat c 1 is a type I hypersensitivity, with the reaction due to IgE (Maciel et al. 2009). IgE-binding epitopes can be classified into discontinuous (conformational) and continuous (sequential) epitopes (Odorico and Pellequer 2003). Conformational IgE epitopes can be destroyed by fragmentation, truncation or reassembly of the molecules, and sequential epitopes are resistant to degradation, even under denaturation, reduction and alkylation conditions (Odorico and Pellequer 2003). To detect continuous epitopes in Jat c 1, we separated the polypeptide chains of this allergen using guanidine hydrochloride and DTT. Mast-cell degranulation assays using the two denatured and isolated Jat c 1 polypeptide chains indicated the presence of continuous IgE epitopes in both the small and large chains (Fig. 4). Allergens belonging to the prolamin superfamily, including 2S albumin seed storage proteins, nonspecific lipid-transfer proteins and cereal α-amylase and protease inhibitors, are present in several important types of allergens of legumes, tree nuts, cereals, fruits and vegetables. These allergens have related structures and are stable under thermal processing and proteolysis, whereas other allergens, including tree pollinosis-associated food allergens such as the Bet v 1 family, have low stabilities under the same conditions (Breiteneder and Radauer 2004).

Mast-cell degranulation induced by synthetic peptides allowed us to identify potential sequential IgE-binding regions in Jat c 1. The twelve peptides spanning the full sequences of both the small and large chains of Jat c 1 were incubated with mast cells previously activated with rat-serum IgE. P1, P4, P5, P6 and P8 promoted high levels of mast-cell degranulation. Interestingly, all of these potential Jat c 1 IgE epitopes contained at least two glutamic acid residues. Experiments with WRK, which modifies glutamic acid, and with a series of related peptides in which strategic glutamic acid residues were substituted by leucine, indicated the importance of carboxylic groups in IgE binding and consequently in mast-cell degranulation. The involvement of glutamic acid in IgE binding was also observed by Denery-Papini et al. (2012), who described a major role of gluten proteins (gliadins and glutenin subunits) in bread making. Their amino acid sequences were unusual, because they contained a high amount of proline (about 30 %) and glutamine (about 40 %) and repetitive sequences. The peptide QPQQPFPQ was one of the repetitive sequences. The substitution of two or three glutamines in this epitope by glutamic acid at positions Q3 or Q4 and Q8 (QPEEPFPE) increased its recognition the most, indicating the importance of glutamic acid in IgE recognition. We used the 3D structure of JHg to localize the potential IgE epitopes of Jat c 1. Potential IgE-binding peptides were located, and P5 and P8, which formed random loops, were the most exposed. These two peptides were consequently deemed to be good candidates for IgE interaction, which occurs when mast cells are exposed to native Jat c 1 protein. The structure of JHg was modeled by Nair et al. (2012), who used as a template Ric c 3, a major allergen from *R. communis*, which was modeled by Pantoja-Uceda et al. (2003). JHg and Ric c 3 are formed by two polypeptide chains linked by disulfide bonds, but their 3D structures were modeled as single polypeptides containing the link peptide that is released by enzymatic cleavage during processing of the 2S albumin precursor. Analysis of the structure of the Jat c 1 precursor, deduced from its DNA sequence, strongly suggests that this precursor is also cleaved after asparagine residues (Fig. 3), similar to the processing of *R. communis* precursor allergens (Irwin et al. 1990). Because P8 would be removed during posttranslational processing, we thus concluded that P5 is a very important region for the stimulation of allergic responses to Jat c 1.

A BLAST search with the Jat c 1 protein sequence found a high degree of homology with *R. communis* allergens, including total conservation of all eight cysteines and nearly all glutamic acid residues. A BLAST search with the sequence of the Jat c 1 large chain found a lower degree of homology with food allergens. The pattern of cysteine residues, however, remained conserved, and this chain contained at least two glutamic acid residues, notably in the section corresponding to P5. The folding pattern allowed by the positioning of the cysteine residues in the Jat c 1 sequence probably contributes to the exposure of the glutamic acid residues, similar to that observed in the *R. communis* allergen. This folding pattern is consistent with the cross-reactivity between Jat c 1 and *R. communis* allergens observed by Maciel et al. (2009). Deus-de-Oliveira et al. (2011) reported cross-reactivity between *R. communis* allergens and wheat, peanut, sesame and gluten allergens. We also found structural homologies between Jat c 1 and these same food allergens and cross-reactivity between Jat c 1 and *R. communis* allergens, so we suggest that Jat c 1 will probably produce an allergenic cross-reaction with these food allergens.

The production of a vaccine against jatropha allergens would be a good strategy for the proper handling of this plant. Our identification of potential IgE binding epitopes can thus contribute to the development of allergen-specific immunotherapies (ASIT). Indeed, knowing the identity of IgE-binding epitopes is essential for the synthesis of hypoallergens employed in the development of such vaccines. Our evidence suggests that, in the case of Jat c 1, the potential IgE-binding epitopes should have their glutamic acid residues substituted by leucine to produce a hypoallergenic protein. The production of minitopes (peptides that block IgE but do not trigger an allergic reaction) from modified Jat c 1 IgE-binding peptides, where the strategic glutamic acid residues could be replaced by leucine residues, is a further ASIT possibility. A similar approach, the modification of critical amino acids in the epitope, was used successfully for the major birch pollen allergen Bet v 1 (Valenta and Niederberger 2007). Finally, the production of vaccines containing “carrier-bound B-cell epitope-containing peptides” is another ASIT strategy Focke-Tejkl et al. (2015). The potential IgE-binding epitopes also can be coupled with viral proteins to prevent an allergic reaction (Chen et al. 2012). Focke-Tejkl et al. (2015) used this approach with Bet v 1 by fusing two nonallergenic peptides and coupling them with the surface protein of hepatitis B. The above strategies are exciting new frontiers to be explored in the future by our group and other researchers worldwide.

## Conclusion

In this study an allergenic protein from *Jatrophacurcas*, Jat c1 was characterized. The IgE-binding peptides and the critical amino acids involved in mast cells degranulation and histamine release were also identified. Since characterization of allergen/IgE-binding is a fundamental step to produce proteins mutant hypoallergenic and vaccine based on peptides, our study contribute to improved efficacy/safety profiles for safer allergy vaccination.

## Additional file

10.1186/s40064-016-2036-5 Synthetic peptides spanning the Jat c 1 sequence.
