# Supplementary material for: Identification of IgE-binding peptide and critical amino acids of Jatropha curcas allergen involved in allergenic response
Source: Springerplus. 2016 Apr 14;5:454. doi: 10.1186/s40064-016-2036-5 (PMC4831952; doi:10.1186/s40064-016-2036-5)
Supplement: Supplementary file 1 — 10.1186/s40064-016-2036-5 Synthetic peptides spanning the Jat c 1 sequence. [file 40064_2016_2036_MOESM1_ESM.pptx]

## Slide 1
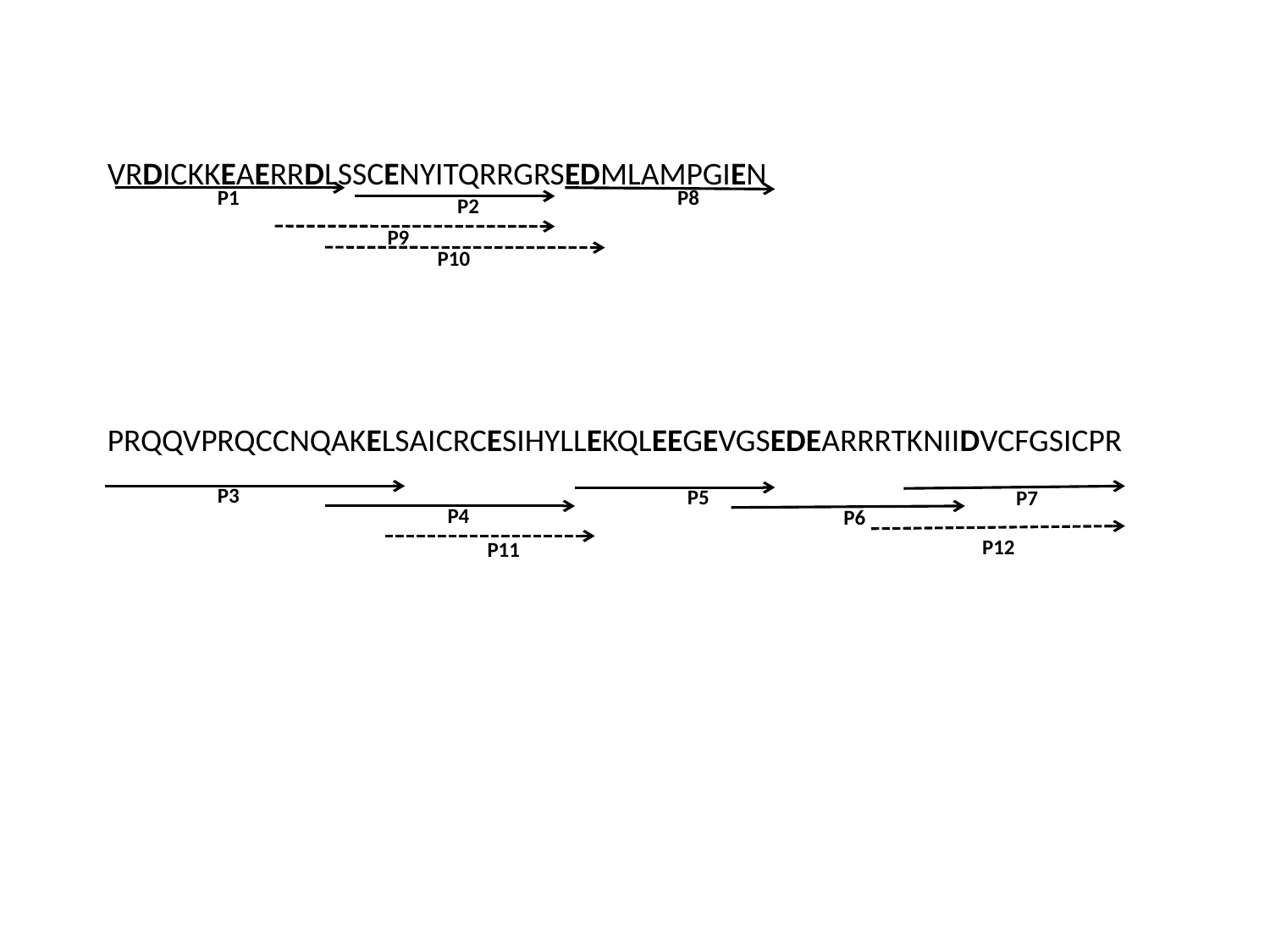

VRDICKKEAERRDLSSCENYITQRRGRSEDMLAMPGIEN
PRQQVPRQCCNQAKELSAICRCESIHYLLEKQLEEGEVGSEDEARRRTKNIIDVCFGSICPR
P1
P8
P2
P9
P10
P3
P5
P7
P4
P6
P12
P11
